# Advanced Metastatic Laryngeal Carcinoma: A Case Report on Palliative Management in Primary Care

**DOI:** 10.7759/cureus.108556

**Published:** 2026-05-09

**Authors:** Susana Couto, Ana Catarina Martins

**Affiliations:** 1 Preventive Medicine, USF Freamunde, ULS de Tâmega e Sousa, Paços de Ferreira, PRT; 2 Family Medicine, USF Freamunde, ULS de Tâmega e Sousa, Paços de Ferreira, PRT

**Keywords:** caregiver burden, laryngeal carcinoma, low health literacy, palliative care, primary health care

## Abstract

This case describes a poorly differentiated laryngeal carcinoma diagnosed at an advanced stage. The disease was associated with extensive metastasis and severe functional impairment. The patient had multiple risk factors, including tobacco and alcohol abuse, low health literacy, and poor therapeutic adherence.

The patient initially presented with dysphonia, followed by progressive cervical swelling. A biopsy confirmed undifferentiated laryngeal carcinoma. Due to clinical deterioration, an emergency tracheostomy was performed. Imaging revealed an extensive infiltrative tumor involving the larynx and hypopharynx, with necrotic lymphadenopathy and distant metastases, including bone and peritoneum. Treatment with cisplatin, 5-fluorouracil, and pembrolizumab was initiated. The clinical course was further complicated by subclavian vein thrombosis.

The patient developed aphonia and severe cervical involvement, with progressive functional decline and increased dependence. Care was ultimately transitioned to the home setting, provided mainly by family members, including a frail primary caregiver.

This case highlights the impact of delayed diagnosis and social vulnerability on disease progression and functional outcomes. It underscores the importance of early recognition, timely intervention, and the central role of primary care-led palliative management and caregiver support in advanced head and neck cancer.

## Introduction

Laryngeal carcinoma is one of the most common malignancies of the head and neck and is strongly associated with tobacco use and chronic alcohol consumption. Early symptoms commonly include dysphonia, odynophagia, and dysphagia. Prognosis largely depends on the tumor stage at diagnosis and is generally favorable in early-stage disease (T1-T2), with five-year survival rates reaching 85-95% following surgery or radiotherapy [1].

Additional risk factors include occupational exposure to certain chemical agents and infection with human papillomavirus (HPV), which has been increasingly associated with some head and neck malignancies [2].

In poorly differentiated tumors or advanced stages (T3-T4), prognosis becomes significantly worse, particularly in the presence of nodal involvement or distant metastasis. Distant metastasis occurs in a small proportion of cases, with the lungs representing the most common metastatic site and being associated with poorer outcomes [3,4]. 

Beyond oncologic progression, laryngeal carcinoma can substantially impair essential functions such as speech, respiration, and swallowing, significantly affecting patients’ quality of life. Psychological distress, including anxiety, depression, and body image disturbances, is frequently reported among patients with head and neck cancers [5,6].

The burden of disease also extends to informal caregivers, typically close family members, who often experience significant emotional stress and caregiving burden throughout the disease trajectory [7,8].

We report the case of a 53-year-old man with a history of heavy tobacco and alcohol use, diagnosed with poorly differentiated metastatic laryngeal carcinoma. This case illustrates the clinical and prognostic challenges associated with advanced-stage disease, as well as its profound impact on functional status, psychological well-being, and caregiver burden. The extensive locoregional metastatic disease significantly impaired his quality of life and contributed to delays in therapeutic management. His elderly mother, with multiple comorbidities and polypharmacy, served as the primary caregiver throughout the disease course.

## Case presentation

A 53-year-old man working as a furniture polisher, from rural northern Portugal, presented in December 2024 for a routine diabetes follow-up with a two-month history of dysphonia. Given the persistence of symptoms and significant risk factors, he was urgently referred to the otorhinolaryngology (ENT) department of a hospital-based healthcare service.

He reported an unintentional weight loss of approximately 5 kg over the past six months, although this was not objectively documented (baseline weight: 63 kg), and a chronic cough, which he attributed to tobacco use. He denied other constitutional or ENT-related symptoms. His medical history included poorly controlled type 2 diabetes mellitus with poor adherence to therapy. He had a significant smoking history (approximately 60 pack-years) and regular alcohol consumption. He remained in the pre-contemplation stage for smoking cessation and repeatedly declined pharmacological or specialized cessation support. His father had died of tobacco-related lung cancer. He lived with his mother.

His regular medication included insulin glargine, telmisartan, rosuvastatin, and ertugliflozin/metformin, with inconsistent adherence.

Given persistent dysphonia for more than eight weeks in a high-risk patient, laryngoscopic examination was performed, revealing an ulcerated exophytic lesion of the left ventricular fold, suspicious for malignancy. The initial histopathological examination was non-diagnostic and revealed a fibrinogranulomatous inflammatory exudate with scattered atypical cells, leading to the recommendation for repeat biopsy. A second biopsy was performed one month after the first procedure, which confirmed a poorly differentiated glottic carcinoma. Following diagnosis, the patient was referred to the Portuguese Oncology Institute for staging and treatment planning.

**Figure 1:**
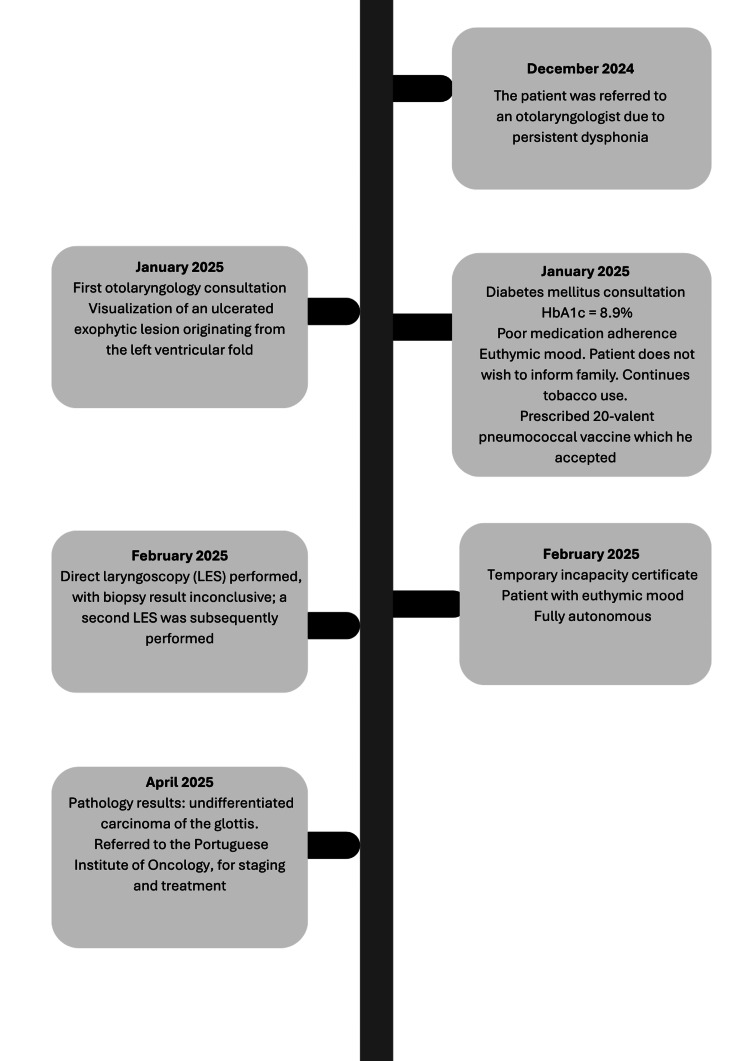
Flowchart of the clinical pathway from hospital referral due to dysphonia to the establishment of the diagnosis. HbA1c: glycated hemoglobin Illustration created by the authors using Canva

Figure 1 illustrates the chronological sequence of major clinical events, including interactions with primary care services.

Staging

Following confirmation of undifferentiated laryngeal carcinoma, the patient was referred to the Portuguese Institute of Oncology (IPO) for further staging.

**Figure 2:**
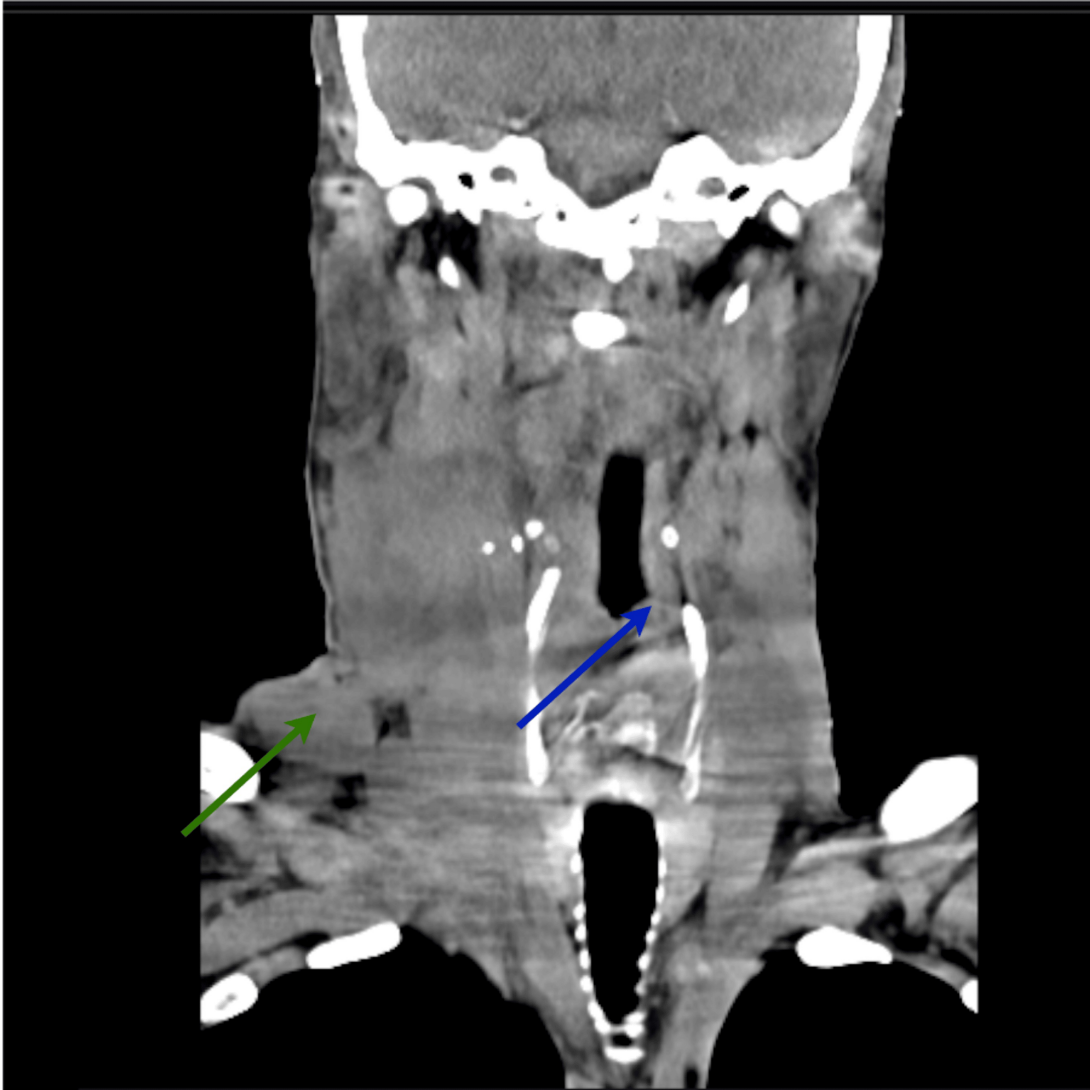
CT scan of the neck, performed in the context of airway obstruction due to a glottic mass, revealed significant narrowing of the laryngeal lumen, invasion of the left laryngeal cartilage, and a conglomerate of cervical lymphadenopathy involving levels II, III, IV, and V, consistent with stage T4N2M1 disease. blue arrow – glottic mass; green arrow – conglomerate of lymph nodes.

On April 30, 2025, he underwent emergency tracheostomy due to airway obstruction and was subsequently hospitalized for a respiratory infection. A CT scan of the neck was performed, which showed severe imaging worsening with an increase in the glottic mass that occupies almost the entire laryngeal lumen with severe airway obstruction, with invasion of the left larynx. At this stage, there was an increase in lymphadenopathy, now with a very prominent right lateral cervical lymph node conglomerate (Figure 2).

After this episode, he was reassessed in primary care, presenting with low mood and difficulty accepting his new clinical condition. He reported right cervical swelling and progressive anorexia. Non-adherence to therapy persisted. He reported smoking cessation following the tracheostomy.

A polyethylene terephthalate (PET) scan performed in May 2025 revealed, in addition to the primary lesion, cervical lymphadenopathy and extensive peritoneal and bone metastases.

Treatment

Systemic therapy with cisplatin, 5-fluorouracil (5-FU), and pembrolizumab was initiated in June. This treatment was started in the context of advanced disease, according to regimens currently recommended in international guidelines for recurrent or metastatic disease, in which the combination of platinum-based chemotherapy and 5-FU with anti-PD-1 immunotherapy (pembrolizumab) has demonstrated a survival benefit in terms of overall survival compared with chemotherapy alone. During this period, the patient developed asymmetric edema of the right upper limb, attributed to tumor-associated thrombosis. CT angiography performed at IPO revealed direct invasion of the right brachiocephalic trunk, with complete thrombotic occlusion of the internal jugular vein extending to the ipsilateral jugular foramen, without opacification of the right transverse sinus. The thrombus also extended distally through the right subclavian and axillary veins, with possible subocclusive thrombi at the cubital fossa and smaller forearm veins.

Imaging further identified a large infiltrative tumor centered in the hypopharynx and larynx, with airway patency maintained via tracheostomy. A necrotic laterocervical lymph node conglomerate was present, involving the submandibular, jugular, and posterior cervical spaces and extending to the supraclavicular region, superior mediastinum, axillary, and suprascapular regions, with invasion of muscular planes and overlying skin. The conglomerate measured 13.8 × 10.2 × 17.4 cm.

In July 2025, the patient was hospitalized due to paraneoplastic malignant hypercalcemia, requiring inpatient stabilization.

**Figure 3:**
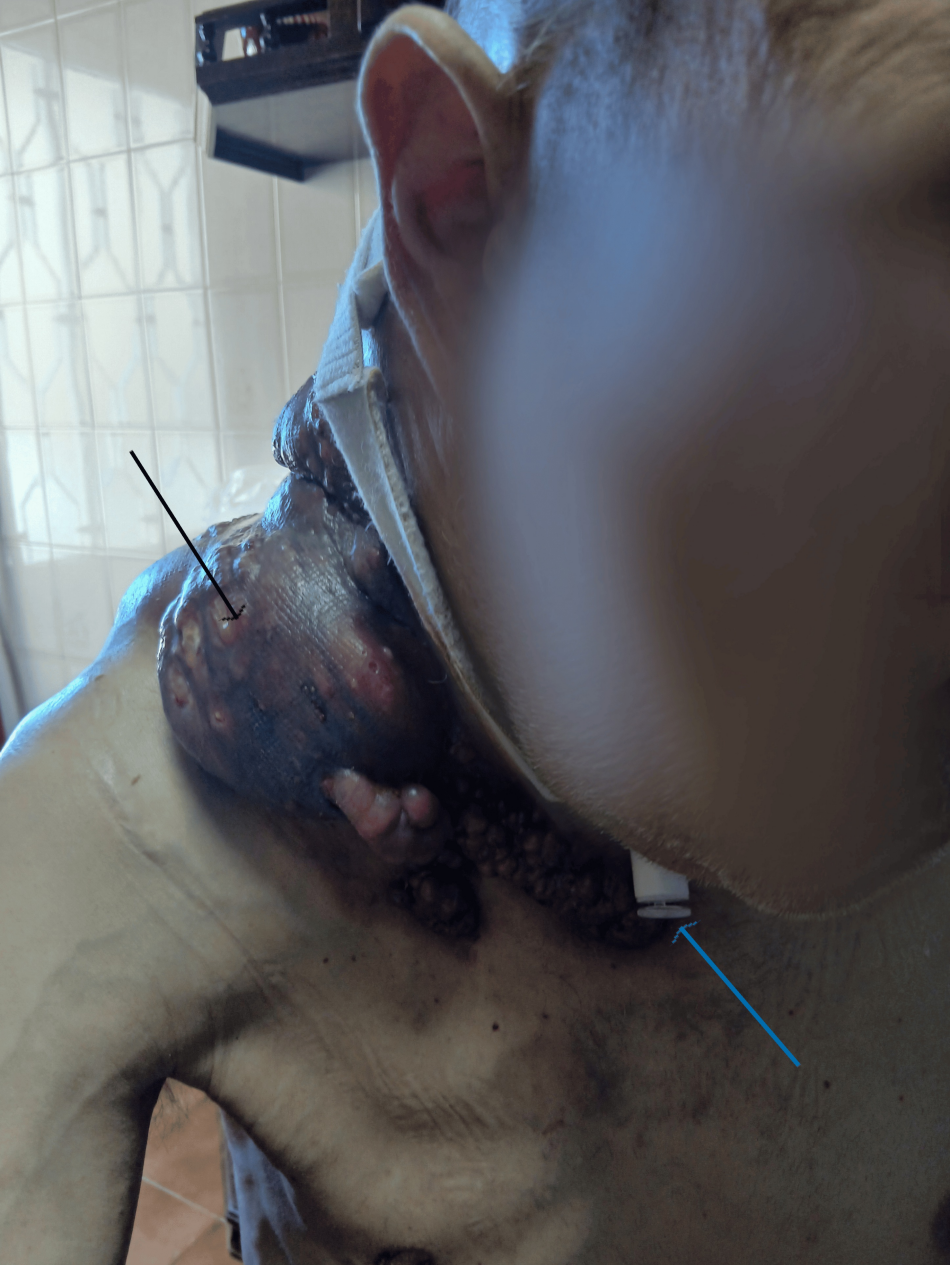
Lymph node conglomerate - anterior view. Black arrow – lymph node conglomerate; blue arrow – tracheostomy cannula.

**Figure 4:**
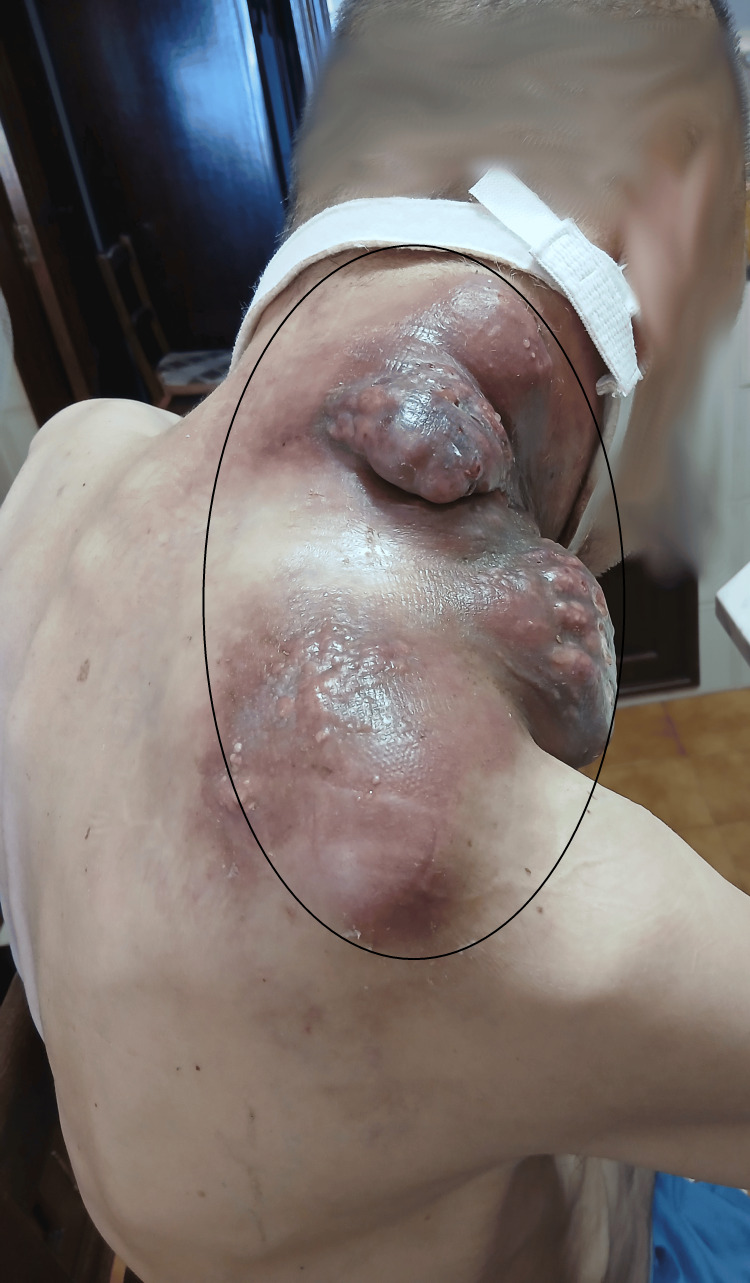
Lymph node conglomerate - posterior view.

Due to extensive cervical tissue invasion, a large tumor mass developed, requiring daily home-based nursing care provided by the Family Health Unit team during the patient’s last month of life at home (Figures 3, 4).

During this period, the patient exhibited significant communication limitations related to the presence of a tracheostomy and required assistance with activities of daily living (ADLs), including feeding, personal hygiene, and dressing. Sleep was fragmented due to persistent cervical discomfort.

Within the scope of home-based palliative care, a multidimensional, patient-centered approach was ensured, including optimization of analgesic therapy in accordance with clinical progression and symptom control. Local care of the lymph node conglomerate was also performed, with dressing changes every two days, ensuring appropriate exudate management and prevention of infectious complications. Cannula replacement was carried out whenever clinically indicated, maintaining patency and patient comfort.

Within the family context, the patient’s mother, the primary caregiver, elderly, widowed, and with multiple comorbidities, demonstrated physical and emotional exhaustion, as assessed by the Zarit Burden Interview. In response, one of the patient’s sisters moved into the household to provide continuous support to both the patient and her mother. Additionally, continuous emotional support was provided to both the patient and the informal caregiver, aiming to promote psychological well-being, facilitate adaptation to disease progression, and support end-of-life care.

This case report was prepared in accordance with the CARE guidelines.

## Discussion

Laryngeal cancer remains one of the most common head and neck malignancies, with tobacco use being the main modifiable risk factor. Tobacco consumption has consistently been identified as the most significant etiological factor, with a dose-response relationship between smoking intensity and cancer risk. Alcohol consumption further amplifies this risk, particularly when combined with tobacco use [1].

Persistent dysphonia lasting more than three weeks in high-risk patients warrants prompt clinical evaluation, as it is often an early symptom of laryngeal malignancy. However, delays in seeking medical attention may result in diagnosis at more advanced stages, negatively affecting outcomes. Population-level data indicate that delayed evaluation of persistent voice symptoms in high-risk patients is associated with reduced overall survival and increased disease progression [9].

This case illustrates a late presentation of laryngeal carcinoma in a patient with multiple risk factors and limited engagement with healthcare services. Despite regular contact with primary care, factors such as persistent tobacco use, poor treatment adherence, and low motivation for behavioral change may have contributed to disease progression and the limitation of therapeutic options. Continued smoking after a diagnosis of head and neck cancer is associated with reduced treatment response, lower disease-free survival, and a higher risk of treatment failure [10,11]. A notable feature of this case was peritoneal metastasis, an extremely rare site of dissemination in laryngeal carcinoma, which typically spreads to the lungs, liver, and bone. Peritoneal involvement is more commonly associated with gastrointestinal or gynaecological malignancies. The few cases reported in the literature describe poor prognosis and limited survival following identification of this metastatic pattern, reinforcing its exceptional nature in head and neck malignancies.

Systemic therapy with cisplatin, 5-fluorouracil (5-FU), and pembrolizumab was initiated in the context of advanced disease, with the aim of disease control and potential survival benefit, in accordance with international recommendations [12]. The choice of this regimen was based on the available evidence and the patient’s clinical context within the available therapeutic options. During the course of the disease, clinical deterioration occurred due to both disease progression and treatment-related complications, leading to discontinuation of systemic therapy and transition to a palliative approach.

In this case, the disease trajectory highlights the importance of timely integration of palliative care. Evidence supports early incorporation of palliative care alongside oncological treatment, improving symptom control, quality of life, and caregiver support, while reducing non-beneficial end-of-life interventions [13]. In contrast, late referrals are associated with poorer symptom control, increased suffering, and greater use of hospital-based care at the end of life.

In this context, the multidisciplinary healthcare team played a central and multifaceted role, with primary care playing a key role in patient follow-up and facilitating transition to palliative care when appropriate. Clinical management, emotional support, patient and family education, and continuous symptom monitoring were integrated to optimize care. Home-based interventions allowed individualized treatment adjustments, effective pain control, and support for dysphagia and aphonia, while reinforcing adherence to symptomatic treatment. This approach illustrates how integrated care can improve quality of life even in advanced disease [14].

The patient progressed to aphonia, functional dependence, and chronic cervical pain, with significant impairment of autonomy and well-being. The impact extended to the family, particularly the mother, who became the main caregiver. Caregivers of patients with advanced cancer often experience a substantial physical, emotional, and social burden, including anxiety, depression, fatigue, and social isolation [15,16].

The healthcare team played an important role in mitigating caregiver burden through home care guidance, emotional support, and communication strategies, ensuring safety and comfort in daily routines. These interventions facilitated family adaptation and preparation for the end-of-life process.

This case highlights the importance of an integrated and multidisciplinary approach involving oncology, palliative care, primary care, and home nursing services. Early intervention targeting modifiable risk factors, reinforcement of health literacy, and compassionate care are essential to support both patients and families throughout the disease trajectory, ensuring symptom control, emotional support, and improved quality of life [1,15,16].

Patient and family perspective

From the family’s perspective, the disease was, from the beginning, interpreted as a direct consequence of the patient’s lifestyle habits, a perception accepted with a certain degree of resignation. Throughout the disease trajectory, the patient rarely expressed his feelings or the emotional impact of the diagnosis and treatment.

He only reported discomfort related to the progressive enlargement of the cervical lymph node mass, which caused physical suffering. His main concern, repeatedly expressed, was the well-being of his family, especially his mother, demonstrating that, more than his own clinical condition, it was the suffering and burden experienced by his relatives that deeply affected him.

Informed consent

Verbal informed consent for the preparation and publication of this case report, including clinical images, was obtained from the patient during follow-up in primary care.

At the time of the written consent request, the patient had significant functional limitation of the right upper limb due to edema and severe pain related to the progression of the cervical tumor. Therefore, with the patient’s full agreement, the consent form was signed by his sister, who lived with the family and was actively involved in his daily care as an informal caregiver.

Throughout the process, the patient’s autonomy, understanding of the information provided, and confidentiality of clinical data were ensured.

## Conclusions

This case highlights how delayed diagnosis, poor treatment adherence, and continued tobacco use led to disease progression, ultimately preventing curative treatment.

This case highlights the important role of multidisciplinary and integrated care teams, including primary care, oncology, palliative care, and home nursing, in addressing complex clinical, functional, and psychosocial needs. Home-based interventions contributed to symptom management, emotional support, and caregiver education. The burden on caregivers highlights the importance of structured support strategies. Early interventions targeting modifiable risk factors, reinforcement of health literacy, and compassionate palliative care may contribute to improved quality of life for both patients and their families.
